# Editorial for the Special Issue: Bioinformatics and Computational Biology for Cancer Prediction and Prognosis

**DOI:** 10.3390/genes16020167

**Published:** 2025-01-28

**Authors:** Garrett M. Dancik, Spiros A. Vlahopoulos

**Affiliations:** 1 Department of Computer Science, Eastern Connecticut State University, Willimantic, CT 06226, USA; 2 First Department of Pediatrics, National and Kapodistrian University of Athens, 11527 Athens, Greece; spirosvlahopoulos2001@gmail.com

Cancer is a global health concern, with approximately 20 million new cancer cases diagnosed worldwide each year. Globally, more than one in nine males and over one in twelve females will die from cancer [1]. As cancer is a genetic disease [2], genomic biomarkers enable the discovery of diagnostic, prognostic, and predictive biomarkers that can lead to personalized cancer treatment. Bioinformatics—which enables the extraction of meaningful information from diverse datasets—plays a vital role in contributing to our understanding of the biological complexity of cancer, yielding insights into more effective treatment. Of utmost importance are tools for data analysis, visualization, and interpretation and their applications that increase our understanding of omics (genomic, transcriptomic, or proteomic) data, as well as bioinformatics tools for processing images and text. Biomarker discovery is aided by data repositories including the Gene Expression Omnibus (GEO) [3] and The Cancer Genome Atlas (*TCGA*) (https://www.cancer.gov/tcga, accessed on 5 January 2025), as well as analytical tools for biomarker identification, such as *CBioportal* [4], *UCSC* Xena [5], *KM Plotter* [6], *shinyGEO* [7] and *ROGUE* [8]. Additionally, databases and tools, such as DAVID [9] and GeneMANIA [10], identify biological functions and pathways associated with one or more genes.

The studies published in this Special Issue, “Bioinformatics and Computational Biology for Cancer Prediction and Prognosis”, provide important insight into a variety of cancers through the use of bioinformatic and computational tools, highlighting the breadth of bioinformatics approaches and tools for cancer research and the diverse types of data (including genomic, proteomic, and image data) being analyzed in order to improve our understanding of cancer biology and to improve treatment.

Breast cancer is the most commonly diagnosed cancer and the leading cause of cancer death in females worldwide [1]. Basmadjian et al. [11] investigated mutations in non-metastatic early-onset breast cancer in 100 women and identified five single-base substitutions and two indel signatures using a non-negative matrix factorization technique. The signatures were named after existing COSMIC signatures if their cosine similarity exceeded a threshold, and the mean relative contribution of each signature was compared against clinical variables. The SBS13-like signature was significantly associated with age group and subtype (HER2-enriched vs. luminal), and the SBSS29-like signature was associated with subtype (TNBC vs. luminal). The SBS13-like signature was also positively associated with recurrence-free survival in a univariate, but not multivariate, analysis. Shiner et al. [12] utilized machine learning models to predict the site of distant metastasis (bone, brain, or visceral) in 175 invasive breast cancer patients, based on clinicopathological variables. An initial multivariate analysis identified ER positivity and N1 stage (compared to N0) as positive predictors of bone metastasis, while HER2 positivity was a negative predictor. Several machine learning models were considered, including naïve Bayes, support vector machines, k-nearest neighbors, and gradient boosting machines. When evaluated on an independent test set, the final models had AUC values of 0.75, 0.74, and 0.73 for predicting bone, brain, and visceral metastasis, respectively.

Li and Xi [13] investigated the RNA expression and function of immune-related genes in cervical squamous cell carcinoma, using a variety of bioinformatics tools. The authors analyzed four microarray datasets and found 186 genes consistently differentially expressed between tumor cells and normal controls, with 22 genes being immune-related according to the ImmPort database [14]. Functional and pathway analysis using DAVID found enrichment in “pathway of signaling mediated by cytokines”, “toll-like receptor signaling pathway”, and “chemokine signaling pathway” among the immune-related genes. Finally, the authors confirmed the differential expression of two genes, *CXCL8* and *CXCL10*, using RT-PCR, identifying promising therapeutic targets in cervical cancer.

Rohan et al. [15] investigated RNA expression differences between patients with low and high *THY1* expression in intestinal gastric cancer, gaining insight into the tumor biology of *THY1* expression. The authors identified 35 genes differentiating patients with low vs. high *THY1* expression, which were validated in multiple cohorts. Subsequent functional analysis found that these genes are significantly associated with biological processes involved in remodeling of the tumor tissue composition, epithelial–mesenchymal transition, and aggressiveness characteristics related to cell motility and migration. In multiple cohorts, high *THY1* expression was associated with poor overall and recurrence-free survival.

Dancik et al. [16] investigated the RNA expression of all 19 aldehyde dehydrogenase (ALDH) genes in acute myeloid leukemia (AML), with the goal of improving AML treatment. The authors found that *ALDH1A1* and *ALDH2* have the strongest association with AML patient risk group classification, and that expression for *ALDH1A1* + *ALDH2* combined has a stronger association with AML patient risk group classification and survival than either gene alone. *ALDH1A1* expression was also found to be higher in recurrent AML. These findings suggest that substances that inhibit the enzymatic activity of both genes are potentially effective pharmaceutics.

Hu et al. [17] investigated tumor-immune-microbe interactions in 422 samples from patients with human papillomavirus-negative head and neck squamous cell carcinoma. The samples were first classified into three groups (hypoxia, mixture, and immune) using a previously published hypoxia-immune classifier, and the authors found that these groups varied with respect to microbial composition. A three-microbial signature was then identified that was predictive of overall survival in the mixture group. The microbial signatures are associated with immune pathways predictive of immunotherapeutic response, which may, therefore, have important implications for treatment selection.

Pan cancer analysis allows for the identification and characterization of biomarkers and pathways common to multiple cancer types or limited to a few [18]. Khan et al. [19] evaluated *TP53* expression across 27 different cancer types, providing a comprehensive evaluation of *TP53* expression with respect to clinical and pathological factors and cancer subtypes. The authors found that *TP53* was overexpressed in a variety of cancers, and *TP53* expression was negatively associated with overall survival in prostate adenocarcinoma. Al-Jarf et al. [20] developed a comprehensive catalogue of non-homologous end joining (NHEJ) DNA repair gene variants in cancer. The final catalog contained 1326 pathogenic and 2390 nonpathogenic missense variants in five genes. Variant analysis was undertaken to statistically identify driver mutations, while a machine learning based predictor was developed, which predicted pathogenic and nonpathogenic variants with 95% and 81% accuracy, respectively. Wu et al. [21] carried out a comprehensive investigation of genes in 150 diseases and developed an integrated pleiotropic gene set consisting of 548 genes associated with five digestive cancers (colorectal, stomach, esophageal, hepatobiliary, and pancreatic). The authors also developed a catalogue of comorbidities for these cancers. The additional identification of hub proteins (*TP53*, *KRAS*, *CTNNB1*, *PIK3CA*) associated with digestive cancers and other diseases can give insight into observed comorbidities, which may impact clinical study design and treatment considerations.

Lastly, information extracted through computed tomography (CT) images, whether through conventional statistical analysis or machine learning, can inform prognosis and treatment. Greco et al. [22] investigated radiogenomic features related to GPTases of immunity-associated proteins (GIMAP) genes from pre-operative CT scans for clear cell renal cell carcinoma patients. The patients were divided into two groups, those with (*n* = 52) or without (*n* = 141) GIMAP expression. There was a significant association between GIMAP expression and several CT features, including the absence of xenophytic growth pattern, tumor infiltration, advanced age, and high Fuhrman grade. Their findings were consistent with tumor inhibition by GIMAPs and suggest that radiogenomic features of GIMAP genes could be used for the development of targeted therapies. Vezakis et al. [23] found that in 40 pancreatic ductal adenocarcinoma (PDAC) patients, pre-operative CT scans were predictive of outcome. Specifically, the authors trained a random forest survival model that had a mean C-index of 0.731 for overall survival and a mean accuracy of 0.76 when predicting two-year survival. The authors used a machine learning approach that automated tumor segmentation and feature extraction, demonstrating the feasibility of a fully automated pipeline to predict survival from pre-operative CT scans.

The studies published in this Special Issue highlight the myriad ways in which bioinformatics can increase our understanding of cancer, using diverse data types (omics data and CT image data) and a variety of methods ranging from traditional approaches for the identification of differentially expressed genes and functional analyses of gene sets to newer approaches such as machine learning methods. As machine learning methods become more mature and more accessible, we expect that the use of more complex methods in bioinformatics, such as neural networks, will increase. However, it is important that machine learning models are developed and used with interpretability in mind. Interpretability yields insight into biological mechanisms [24] and may be required for the clinical use of bioinformatics tools.

Important challenges in bioinformatics remain. As in other fields such as the social sciences [25], cancer research findings cannot always be replicated [26]. While non-computational factors play a role, bioinformatics research should follow best practices for computational reproducibility, including version control for code and persistent access to code and data [27]. Reproducible workflows should also be developed and used [28]. It is exciting to be a researcher during a time when large amounts of cancer data are readily available and where recent advances in artificial intelligence have made coding and data analysis more accessible. While data exploration is essential, exploratory approaches need to be clearly disclosed, and care must be taken to limit the number of false positives by following appropriate frameworks [29]. Finally, bioinformatics has always been a predictive science. The studies in this field make many predictions regarding the importance of genes, proteins, or other clinical characteristics in a variety of cancers. In many cases, though, evaluation in prospective studies and/or experimental validation are needed to confirm the validity of the findings. Experimental validation is expensive, time-consuming, and not always feasible for computational researchers, but advances in AI and robotics will allow for the assistance and automation of laboratory experiments, once AI reaches the level of maturity that gives it a minimum degree of reliability and reproducibility [30]. A more comprehensive computational pipeline—for both data analysis using bioinformatics and experimental validation aided by robotics—will more quickly lead to improvements in cancer related care.

As the guest editors of this Special Issue, we would like to thank all the authors and reviewers who contributed.
